# Clinical factors influencing the impact of cluster headache from a prospective multicenter study

**DOI:** 10.1038/s41598-020-59366-9

**Published:** 2020-02-12

**Authors:** Jong-Hee Sohn, Jeong-Wook Park, Mi Ji Lee, Pil-Wook Chung, Min Kyung Chu, Jae Myun Chung, Jin-Young Ahn, Byung-Su Kim, Soo-Kyoung Kim, Yun-Ju Choi, Daeyoung Kim, Tae-Jin Song, Kyungmi Oh, Heui-Soo Moon, Kwang-Yeol Park, Byung-Kun Kim, Dae-Woong Bae, Chin-Sang Chung, Soo-Jin Cho

**Affiliations:** 1 Department of Neurology, Chuncheon Sacred Heart Hospital, Hallym University College of Medicine, Chuncheon-si, Korea; 2 Department of Neurology, Uijeongbu St.Mary’s Hospital, The Catholic University of Korea College of Medicine, Uijeongbu, Korea; 3 Department of Neurology, Neuroscience Center, Samsung Medical Center, Sungkyunkwan University School of Medicine, Seoul, Korea; 4 Department of Neurology, Kangbuk Samsung Hospital, Sungkyunkwan University School of Medicine, Seoul, Korea; 5 Department of Neurology, Severance hospital, Yonsei University College of Medicine, Seoul, Korea; 6 Department of Neurology, Seoul Paik Hospital, Inje University College of Medicine, Seoul, Korea; 7 Department of Neurology, Seoul Medical Center, Seoul, Korea; 8 Department of Neurology, Bundang Jesaeng General Hospital, Daejin Medical Center, Seongnam, Korea; 9 Department of Neurology, Gyeongsang National University College of Medicine, Jinju, Korea; 10 Department of Neurology, Presbyterian Medical Center, Jeonju, Korea; 11 Department of Neurology, Chungnam National University College of Medicine, Daejeon, Korea; 12 Department of Neurology, Mokdong Hospital, College of Medicine, Ewha Womans University, Seoul, Korea; 13 Department of Neurology, Korea University College of Medicine, Seoul, Korea; 14 Department of Neurology, Chung-Ang University Hospital, Chung-Ang University College of Medicine, Seoul, Korea; 15 Department of Neurology, Eulji Hospital, Eulji University, Seoul, Korea; 16 Department of Neurology, College of Medicine, The Catholic University of Korea, Suwon, Korea; 17 Department of Neurology, Dongtan Sacred Heart Hospital, Hallym University College of Medicine, Hwaseong, Korea

**Keywords:** Headache, Risk factors

## Abstract

Although many patients with cluster headaches (CH) are disabled by their condition, few studies have examined this in detail. This cross-sectional, multicenter observational study prospectively collected demographic and clinical questionnaire data from 224 consecutive patients with CH. We assessed headache impact using the six-item Headache Impact Test (HIT-6) and evaluated the factors associated with the impact of CH. Participants with a HIT-6 score ≥ 60 were classified into a severe impact group. The majority (190, 84.8%) of the participants were classified into the severe impact group. These patients were characterized by younger age, earlier onset of CH, longer duration of each headache attack, higher pain intensity, more cranial autonomic symptoms, a higher proportion of depression or anxiety, higher score of stress, and lower score of quality of life. The anxiety (OR = 1.19, 95% CI: 1.08–1.31, *p* = 0.006), greater pain intensity (OR = 1.06, 95% CI: 1.02–1.10, *p* = 0.002), and age (OR = 0.99, 95% CI: 0.99–1.00, *p* = 0.008) were significant predictors for a severe impact of CH patients. According to the HIT-6 results, most of the CH patients were significantly affected by CH. As well as pain intensity, anxiety and age modulated CH’s impact on their lives.

## Introduction

Cluster headache (CH) is one of the most painful and disabling primary headache disorders, but the severity and rate of disability have not been fully assessed using a headache-specific tool. Headache-related disability is an important factor in the treatment of headache disorders and can help determine the success of treatment regimens^1,2^. The impact of recurrent headaches is best explained by three factors: pain density (representing headache activity), affective distress (psychiatric comorbidity), and disability (social functioning and work efficacy)^3^. Headache-specific tools used to measure headache-related disability include the Headache Disability Inventory^4^, Headache Impact Questionnaire (HImQ)^5,6^, Migraine-specific Quality of Life Questionnaire^7^, Migraine Disability Assessment Scale (MIDAS)^8^, and six-item Headache Impact Test (HIT-6)^9^. The HIT-6 is based on items taken from the Headache Disability Inventory, Headache Impact Questionnaire, MIDAS, and Migraine-specific Quality of Life Questionnaire and measures the overall impact of headache on the patient’s life^10,11^. The HIT-6 has been applied to more than 1,000 subjects in the United States, and its validity and reliability for assessing mild, moderate, and severe headaches have been confirmed^12–15^. Unlike the MIDAS, which focuses on migraine patients and assesses disability related to daily life, the HIT-6 can be applied to a variety of headache disorders to measure their impact over a wide range of domains. The HIT-6 score is associated with quality of life measures^11,16,17^, and headache severity affects the HIT-6 score^16,18,19^. The HIT-6 score is also influenced by psychiatric comorbidities^20,21^. Tests that assess the impact of headache may be affected by accompanying clinical factors, but these effects are not yet clear.

Some studies have examined the impact of CH on quality of life and showed reduced wellbeing in many quality of life domains^22–24^ and increased disability due to the headaches^25–27^. The level of quality of life impairment in CH patients is similar to that in migraine patients^23^, whereas the disability due to headache is higher in CH than migraine patients^26^. Although disability has been widely examined in migraine patients, relatively little is known about disability in patients with CH. In addition, the clinical factors associated with disability in CH patients have rarely been reported. Therefore, this study investigated the extent of disability in a CH cohort in Korea using the HIT-6. We hypothesized that the majority of patients with CH are severely affected by the disorder and thus examined the clinical factors associated with a severe impact of CH.

## Methods

### Study design and population

This study was based on the multicenter, cross-sectional Korean Cluster Headache Registry (KCHR) and used prospectively collected data from consecutive patients with CH treated at neurology outpatient departments in Korea between September 2016 and December 2018. Sixteen hospitals participated in the KCHR: 14 university hospitals (8 tertiary and 6 secondary referral hospitals) and 2 general hospitals. This study investigated the demographic and clinical features and extent of disability of Korean CH patients aged 19 years or older. The KCHR data have been partially analyzed and a detailed description of the study process described previously^28–30^. All participants were examined by each investigator to confirm that the diagnosis met the criteria for CH of the International Classification of Headache Disorders, third edition (ICHD-3, beta version) and were asked to complete a questionnaire^31^. The ICHD-3 was published after the initial recruitment phase of the KCHR, so the participants were re-diagnosed using the ICHD-3 based on each patient’s clinical history; those who did not meet the criteria for CH were excluded from this analysis^30^.

Written informed consent was obtained from all study participants before entering the study. This study protocol was approved by each participating hospital: Dongtan Sacred Heart Hospital (2016-396-I), Samsung Medical Center (2016-09-123), Uijeongbu St. Mary’s Hospital (XC16OIMI0087U), Gyeongsang National University College of Medicine (2016-10-022-001), Korea University College of Medicine (KUGH16315), Bundang Jesaeng General Hospital (NR16-04), Seoul Medical Center (2016-013), Kangbuk Samsung Hospital (KBSMC2016-11-032), Eulji University School of Medicine (2016-11-004), Ewha Womans University School of Medicine (2016-09-021), Seoul Paik Hospital (PAIK 2016-11-003), Presbyterian Medical Center (2016-10-046), Chungnam National University College of Medicine (CNUH 2017-12-046), Chuncheon Sacred Heart Hospital (2016-116), Severance Hospital (4-2018-0511), and the Catholic University of Korea (2018-3145-0001). Chung-Ang University Hospital (1621-002-264) did not enroll any participants after the study was approved and therefore was not counted as a participating hospital. All investigations were conducted according to the tenets of the Declaration of Helsinki.

### Clinical information and questionnaire

The KCHR protocol evaluated sociodemographic variables, including sex, ages at onset and presentation, body mass index, and histories of smoking and alcohol consumption. Investigators collected the following clinical data regarding current and previous CH periods: severity of pain on a numeric rating scale, duration and frequency of headache attacks, average duration of a CH bout, and total CH periods.

The impact of headaches was assessed using the HIT-6^12^. The HIT-6 score is obtained by summing the individual scores for each of its six items. The total score ranges from 36 to 78, and a higher score reflects a greater impact from the headaches. Each item is scored using a frequency scale and is assigned an item category weight (6, 8, 10, 11, or 13). Headache impact severity was categorized as follows, based on the HIT-6 interpretation scoring guide: little or no impact (≤49), some impact (50–55), substantial impact (56–59), and severe impact (60–78). The patients were divided into two subgroups: CH with a severe impact (CH + S; HIT-6 score ≥ 60) and CH without a severe impact (CH–S; HIT-6 score ≤ 59). Demographic and clinical variables were compared between these two groups. Each patient completed the self-administered EQ-5D index (EuroQol) as a measure of health-related quality of life^32^. Depression, anxiety, and psychological stress were assessed using the Patient Health Questionnaire-9 (PHQ-9), Generalized Anxiety Disorder-7 (GAD-7) scale, and Short Form Perceived Stress Scale (PSS-4), respectively^33–35^. Cut-off points for depression and anxiety were defined as a PHQ-9 score ≥8 and GAD-7 score ≥6, respectively. The Cluster Headache Severity Scale (CHSS) was recently introduced and used in a Swedish cohort^36^, and we also applied it in our population. The CHSS assesses three factors (CH attack duration, number of attacks per day, and duration of CH bouts), and the total score ranges from 3 to 12.

### Statistical analysis

This study was based on prospective registry data, and we analyzed all available data. Therefore, the sample size was calculated to verify the validity of the statistical analysis. The calculated sample size of each group was 34 in the analysis of age differences (delta = 7, standard deviation = 10, significance level = 0.05, and power = 0.8).

Descriptive statistics are presented as means  ±  standard deviation, medians (25^th^, 75^th^ percentiles), or numbers (percentages). After confirming the normality of data distributions using the Kolmogorov−Smirnov test, we used independent *t*-tests to compare continuous variables and Fisher’s exact test to compare categorical variables between the two groups (CH + S and CH − S). If the distribution was not normal, the Mann−Whitney *U-*test was used.

The information value (IV) statistic was calculated for the variables that differed significantly between the two groups (CH + S and CH − S). IV is mainly used to reduce the number of variables in the initial step of a logistic regression analysis^37^. An IV ≥ 0.3 is the criterion for selecting independent variables. After selecting the variables, the univariable and multivariable logistic regression analyses were used to identify predictors of a severe impact of CH. All tests were two-tailed, and a *p*-value < 0.05 was considered to represent statistical significance. All analyses were performed using R for Windows (ver. 3.6.1; R Foundation for Statistical Computing, Vienna, Austria) and RStudio (ver. 1.1.442; RStudio, Boston, MA, USA).

## Results

This study prospectively enrolled 250 CH patients from September 2016 to December 2018. We excluded 25 patients who responded during a remission period and 1 patient who did not complete the HIT-6. Of the 224 remaining patients, 29 (12.9%) were experiencing their first episode of CH, 158 (70.5%) had episodic CH, 11 (4.9%) had chronic CH, and 26 (11.6%) had probable CH according to the ICHD-3 criteria. The mean age at the time of investigation was 38.3 ± 10.8 years (males, 38.1 ± 10.2; females, 39.4 ± 13.8), and 189 (84.4%) of the 224 patients were male.

### Impact of CH according to the HIT-6 score

**Figure 1:**
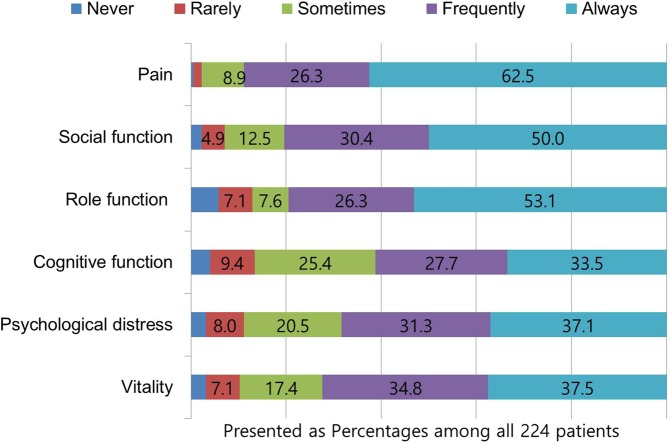
Distribution of responses to individual HIT-6 items of 244 patients with cluster headache.

The HIT-6 scores of the CH patients ranged from 41 to 78, with a mean score of 68.5 ± 7.9 (males, 68.2 ± 8.1; females, 69.6 ± 6.9; *p* = 0.345). Regarding the impact of CH, 3 (1.3%) patients reported little or no impact, 13 (5.8%) reported some impact, 18 (8.0%) reported substantial impact, and 190 (84.8%) reported severe impact. The HIT-6 score of the patients differed according to the CH subtype (first episode of CH, 66.6 ± 6.8; episodic CH, 69.3 ± 7.6; chronic CH, 70.9 ± 4.6; and probable CH, 64.1 ± 10.5; *p* = 0.006). In the *post-hoc* analysis using Tukey’s test, the HIT-6 score was lower for the patients with probable CH than those with chronic CH (*p* = 0.009), but there were no differences among the other groups. In the analysis of individual HIT-6 items, the proportions of participants who responded that “headache attack always affected [a particular domain]” were as follows: pain, 62.5%; role function, 53.1%; social functioning, 50.0%; vitality, 37.4%; psychological distress, 37.1%; and cognitive function, 33.5% (Fig. 1).

### Comparison of clinical factors between CH with and CH without a severe impact

Comparisons of the demographic and clinical variables between the CH + S (n = 190) and CH − S (n = 34) groups are presented in Table 1. The CH + S group was significantly younger (median 36.0 *vs*. 43.0 years, *p* < 0.001) and had an earlier onset of CH illness (median 26.5 *vs*. 36.0 years, *p* < 0.001) compared with the CH − S group. The CH + S group had longer individual headache attacks (median 90 *vs*. 60 min, *p* = 0.018), greater pain intensity on a visual analogue scale (median 9.0 *vs*. 8.0, *p* = 0.001), and more cranial autonomic symptoms (median 4.0 *vs*. 2.5, *p* = 0.002) compared with the CH − S group. Sex and other clinical parameters did not differ between the two groups.

**Table 1:** Comparison of clinical features between CH patients with and without severe headache impact.

	CH + S (n = 190)	CH − S (n = 34)	Total CH (n = 224)	*P-*value
Age, years	36.0 [30.0;42.0]	43.0 [40.0;50.0]	37.0 [30.8;44.0]	<0.001
Onset age of CH, years	26.5 [19.0;34.0]	36.0 [30.0;43.0]	28.5 [19.8;36.0]	<0.001
Female sex, n. (%)	31 (16.3)	4 (11.8)	35 (15.6)	0.677
BMI, kg/m^2^	24.2 ± 3.1	23.8 ± 2.6	24.1 ± 3.1	0.449
Previous migraine history, n. (%)	28 (14.7)	3 (8.8)	31 (13.8)	0.433
Current smoking, n (%)	80 (42.1)	17 (50.0)	97 (42.4)	0.504
Current alcohol consumption, n (%)	87 (45.81)	17 (50.0)	95 (54.3)	0.789
Chronic CH (%)	11 (5.8)	0 (0)	11 (4.9)	0.313
Recurrence, n (%)	153 (80.5)	25 (73.5)	178 (79.4)	0.484
Diurnal variation, n (%)	104 (54.7)	20 (58.8)	124 (55.3)	0.799
Seasonal variation, n (%)	77 (46.1)	14 (45.2)	91 (45.9)	1.000
Total period of CH, years	7.0 [2.0;14.0]	5.0 [1.0;12.0]	6.5 [2.0;14.0]	0.297
Average duration of CH bout, week	4.0 [3.0; 8.0]	4.0 [1.4; 6.0]	4.0 [2.4; 6.0]	0.130
Attack frequency per day	1.5 [1.0; 3.0]	1.5 [1.0; 2.0]	1.5 [1.0; 3.0]	0.595
Attack duration, minutes	90.0 [60.0;120.0]	60.0 [45.0;90.0]	80.0 [60.0;120.0]	0.018
Headache intensity, VAS	9.0 [8.0;10.0]	8.0 [8.0;10.0]	9.0 [8.0; 10.0]	0.001
Number of autonomic symptoms	4.0 [2.0; 5.0]	2.5 [1.0; 4.0]	4.0 [2.0; 5.0]	0.002
Presence of restless/agitation, n (%)	95 (50.0)	12 (35.3)	107 (47.8)	0.163
CHSS score	6.0 [5.0; 6.0]	5.0 [5.0; 6.0]	5.0 [5.0; 6.0]	0.027
Depression (PHQ-9 ≥ 8), n (%)	79 (41.8)	6 (17.6)	85 (38.1)	0.013
Anxiety (GAD-7 ≥ 6), n (%)	129 (68.6)	10 (29.4)	139 (62.59)	<0.001
Quality of life (EQ-5D)	0.87 [0.75;0.91]	0.91 [0.88;1.00]	0.90 [0.77;1.00]	<0.001
Stress severity (PSS-4)	7.0 [5.0;9.0]	5.5 [4.0;8.0]	7.0 [5.0;8.0]	0.011

Presented as median [25%, 75%] or mean ± standard deviation; CI, confidence interval; CH, cluster headache; CH − S, cluster headache without severe impact; CH + S, cluster headache with severe impact; BMI, body mass index; VAS, visual analogue scale; CHSS, Cluster Headache Severity Scale; PHQ-9, Patient Health Questionnaire-9; GAD-7, Generalized Anxiety Disorder-7 scale; EQ-5D, EuroQol EQ-5D index; PSS-4, Short Form Perceived Stress Scale, NA, not available due to one group was empty.

The proportions of patients with anxiety (GAD-7 score ≥ 6, 68.6% *vs*. 29.4%, *p* < 0.001) and depression (PHQ-9 score ≥ 8, 41.8% *vs*. 17.6%, *p* = 0.013) were also higher in the CH + S group. Quality of life was significantly lower in the CH + S group, according to the median EQ-5D score (0.87 *vs*. 0.91, *p* < 0.001), and the median CHSS score (6.0 *vs*. 5.0, *p* = 0.027) and median PSS-4 score (7.0 *vs*. 5.5, *p* = 0.011) was significantly higher in the CH + S group.

### Univariable and multivariable regression logistic analyses of severe headache impact

The IV of the significant variables in Table 1 were as follows: anxiety (GAD-7 ≥ 6), 0.65; age, 0.53; headache intensity, 0.53; age of CH onset, 0.47; stress severity, 0.45; quality of life, 0.34; depression (PHQ-9 ≥ 8), 0.29; number of autonomic symptoms, 0.29; CHSS score, 0.27; CH attack duration, 0.20; and presence of restlessness/agitation, 0.09. Therefore, we included these variables with IV ≥ 0.3 in the regression analyses. Table 2 shows the results of the univariable and multivariable logistic regression analysis performed to identify predictors associated with the CH + S group. In the logistic analyses, all included variables were significantly associated with a severe impact of headache. In the multivariale logistic analysis, presence of anxiety (OR = 1.19, 95% CI: 1.08–1.31, *p* = 0.006), greater pain intensity (OR = 1.06, 95% CI: 1.02–1.10, *p* = 0.002), and age (OR = 0.99, 95% CI: 0.99–1.00, *p* = 0.008) remained significantly associated with a severe impact of CH.

**Table 2:** Univariable and multivariable logistic regression model of the factors associated with severe impact of headaches.

	Univariable Analysis		Multivariable Analysis	
	*P-*value	Odds ratio (95% CI)	*P-*value	Odds ratio (95% CI)
Age	<0.001	0.99 (0.99–1.00)	0.008	0.99 (0.99–1.00)
Onset Age	<0.001	0.99 (0.99–1.00)	0.661	1.00 (0.99–1.00)
Headache intensity, VAS	<0.001	1.07 (1.03–1.11)	0.002	1.06 (1.02–1.10)
Anxiety (GAD-7 ≥ 6)	<0.001	1.24 (1.13–1.37)	0.006	1.19 (1.08–1.31)
Quality of life (EQ-5D)	0.003	0.71 (0.57–0.89)	0.325	0.88 (0.69–1.13)
Stress severity (PSS-4)	0.015	1.02 (1.00–1.04)	0.426	1.01 (0.99–1.02)

CI, confidence interval; VAS, visual analogue scale; GAD-7, Generalized Anxiety Disorder-7 scale; EQ-5D, EuroQol 5D index; PSS-4, Short Form Perceived Stress Scale.

## Discussion

In this KCHR-based study, the majority (84.8%) of participants were classified as CH + S according to their HIT-6 score. This group was characterized by a younger age, earlier onset of CH illness, longer duration of individual headache attacks, greater pain intensity, higher stress score, lower score in the quality of life, and more cranial autonomic symptoms compared with the CH − S group. The proportions of patients with depression and anxiety were also greater in the CH + S group. Multivariable logistic regression analysis showed that anxiety, younger age, and greater pain intensity were significant predictors of a severe impact of CH.

We investigated the degree of disability caused by CH by examining the patients’ ability to perform daily living activities using the HIT-6 and found no significant difference between chronic and episodic CH patients. Although not directly comparable, one study reported that the impact of headache was greatest in chronic CH patients (n = 27), followed by those currently experiencing a bout of episodic CH (n = 26), those currently in remission from episodic CH (n = 22), and those with migraine^27^. One study found no significant difference in the HIT-6 score between 11 patients with episodic CH (64.60 ± 4.81) and 11 patients with chronic CH (64.60 ± 11.86)^24^, similar to our findings. In contrast, another study reported higher HIT-6 scores in 72 patients with chronic CH (61.78 ± 8.05) compared with 107 patients with episodic CH (53.25 ± 7.57)^38^. Our participants were assessed during a CH bout, and thus the findings indicate that the disability caused by episodic CH (69.3 ± 7.6) was as severe as that caused by chronic CH (70.9 ± 4.6). Probable CH is reported as having a similar impact to definite CH, while probable and chronic CH have not been compared^30^. The impact of probable CH should be re-evaluated.

In our study, the CH + S patients were younger and had earlier-onset CH compared with the CH − S group. In line with our results, a previous study reported that CH at a younger age had a greater impact on the personal and professional lives of the patient^24^. Another study reported that depressed or anxious CH patients were more likely to present for treatment at a younger age and to have prodromal symptoms^39^. These results suggest the importance of the influence of personal and demographic characteristics on the presentation of CH patients and their ability to cope with their illness.

Psychiatric comorbidities commonly reported in patients with CH include anxiety, depression, bipolar disorder, and suicidality^40–42^. In cross-sectional studies, the incidence of anxiety ranged from 11.8% to 75.7% and that of depression from 6.3% to 43%^40^. Similarly, we found that anxiety was common in CH patients and was a significant predictor of severe disability. Agitation/restlessness during CH may influence anxiety during a CH period. One study investigated 13 episodic CH patients who underwent ^18^F‐fluorodeoxyglucose positron emission tomography and found that the extent of disability was correlated with glucose metabolism in the amygdala^43^. Amygdala activity is reportedly enhanced in anxiety disorder, so the association between anxiety and severe disability may be plausible in a severe pain syndrome, such as CH^44^. Considering these clinical observations, CH patients may experience severe disability if their disease is accompanied by psychiatric disorders.

Unlike CH attack duration or pain intensity, the number of attacks per day was not correlated with the HIT-6 scores. Several explanations are possible. First, the number of attacks per day can vary during a cluster period, so the recent frequency did not reflect the impact of the entire cluster period. Second, attack frequency can be influenced by the attack duration, similar to the higher frequency related to a shorter duration in other trigeminal autonomic cephalalgias. The attack duration in CH patients who reported two or fewer attacks per day was slightly longer than that in those with more frequent attacks (109.7 ± 128.3 *vs*. 93.2 ± 67.3 minutes, *p* = 0.235) in this study. This suggests that attack duration or pain intensity should be considered when assessing the efficacy of preventive medication in CH.

Recently, a new index, the CHSS, was proposed as a useful tool for clinically classifying recently diagnosed CH patients^36^. The CHSS focuses on daily headache attack frequency and the durations of individual attacks and bouts. In our study, the mean CHSS score was significantly higher in the CH + S than in the CH − S group. This suggests that the durations of the individual attacks and bouts are important in terms of the extent of the disability associated with CH. Unlike the CH index, which is calculated as (attacks per day × hours per attack) × (days per clusters × clusters per year)^45^, the CHSS does not consider the number of clusters per year, which can vary. The HIT-6 assesses headache attacks over the past 4 weeks. In CH, headache intensity appears to influence the HIT-6 score, whereas the CHSS is influenced more by headache frequency and duration. Therefore, used together, these scales may provide a more accurate assessment of the extent of a patient’s CH-related disability.

There were several limitations to this study. First, all of the subjects were treated in secondary or tertiary headache centers, which might be associated with selection bias. Second, there was an insufficient number of chronic CH patients to generalize the results in this population. In Asia, the proportion of headache sufferers with chronic CH is relatively low (0–7.5%), and the clinical profiles of patients with chronic CH have not been evaluated separately from those of patients with episodic CH^46–48^. Third, the HIT-6 questionnaire is a self-report instrument with the potential for recall bias. Assessing both HIT-6 results and headache frequency after treatment might be helpful for judging the efficacy of CH treatment.

## Conclusions

According to the HIT-6 scores, most of our CH patients were severely affected by their condition, and the impact of episodic CH was as severe as that of chronic CH. This study identified anxiety, younger age, and greater pain intensity as significant predictors of a severe impact of CH. These results emphasize the importance of early diagnosis of CH patients suffering from severe effects starting at a younger age. Future research should examine whether treating anxiety may help reduce the impact of headache.

## Data Availability

Public sharing of the data set used in this study is restricted by an IRB of participating hospital (the Institutional Review Board of Samsung Medical Center). To request the data, readers should contact to Research department of Korean Headache Society (http://www.headache.or.kr/index.php, kheadache2014@gmail.com).
